# Lyapunov-type inequalities for an anti-periodic fractional boundary value problem involving *ψ*-Caputo fractional derivative

**DOI:** 10.1186/s13660-018-1850-4

**Published:** 2018-10-20

**Authors:** Bessem Samet, Hassen Aydi

**Affiliations:** 1 Nonlinear Analysis Research Group, Ton Duc Thang University, Ho Chi Minh City, Vietnam; 2 Faculty of Mathematics and Statistics, Ton Duc Thang University, Ho Chi Minh City, Vietnam; 3 Department of Mathematics, College of Education of Jubail, Imam Abdulrahman Bin Faisal University, Jubail, Saudi Arabia; 4 Department of Medical Research, China Medical University Hospital, China Medical University, Taichung, Taiwan

**Keywords:** 34A08, 26D10, 34L15, Lyapunov-type inequalities, anti-periodic fractional boundary value problem, *ψ*-Caputo fractional derivative, eigenvalues

## Abstract

A Lyapunov-type inequality is established for the anti-periodic fractional boundary value problem

$$\begin{aligned} & \bigl({}^{C}D_{a}^{\alpha,\psi}u \bigr) (x)+f \bigl(x,u(x) \bigr)=0,\quad a< x< b, \\ &u(a)+u(b)=0,\qquad u'(a)+u'(b)=0, \end{aligned}$$

where $(a,b)\in\mathbb{R}^{2}$, $a< b$, $1<\alpha<2$, $\psi\in C^{2}([a,b])$, $\psi'(x)>0$, $x\in[a,b]$, ${}^{C}D_{a}^{\alpha,\psi}$ is the *ψ*-Caputo fractional derivative of order *α*, and $f: [a,b]\times\mathbb{R}\to\mathbb{R}$ is a given function. Next, we give an application of the obtained inequality to the corresponding eigenvalue problem.

## Introduction

In this paper, we are concerned with the anti-periodic fractional boundary value problem

1.1 $$ \begin{aligned} & \bigl({}^{C}D_{a}^{\alpha,\psi}u \bigr) (x)+f \bigl(x,u(x) \bigr)=0,\quad a< x< b, \\ &u(a)+u(b)=0,\qquad u'(a)+u'(b)=0, \end{aligned} $$

where $(a,b)\in\mathbb{R}^{2}$, $a< b$, $1<\alpha<2$, $\psi\in C^{2}([a,b])$, $\psi'(x)>0$, $x\in[a,b]$, ${}^{C}D_{a}^{\alpha,\psi}$ is the *ψ*-Caputo fractional derivative of order *α*, and $f: [a,b]\times\mathbb{R}\to\mathbb{R}$ is a given function. A Lyapunov-type inequality is derived for problem (1.1). Next, as an application of the obtained inequality, an upper bound of possible eigenvalues of the corresponding problem is obtained.

Let us mention some motivations for studying problem (1.1). Suppose that $u\in C^{2}([a,b])$, $(a,b)\in\mathbb{R}^{2}$, $a< b$, is a nontrivial solution to the boundary value problem

1.2 $$ \begin{aligned} &u''(x)+w(x)u(x)=0, \quad a< x< b, \\ &u(a)=0,\qquad u(b)=0, \end{aligned} $$

where $w\in C([a,b])$ is a given function. Then (see [17])

1.3 $$ \int_{a}^{b} \bigl\vert w(x) \bigr\vert \,dx > \frac{4}{b-a}. $$

Inequality (1.3) is known in the literature as Lyapunov’s inequality, which provides a necessary condition for the existence of a nontrivial solution to (1.2). Many generalizations and extensions of (1.3) were derived by many authors. In particular, Hartman and Wintner [9] proved that if $u\in C^{2}([a,b])$ is a nontrivial solution to (1.2), then

1.4 $$ \int_{a}^{b} (b-s) (s-a) w^{+}(s)\,ds>b-a, $$

where

$$w^{+}(s)=\max \bigl\{ w(s),0 \bigr\} ,\quad a\leq s\leq b. $$

It can be easily seen that (1.3) follows from (1.4). For other results related to Lyapunov-type inequalities, see, for example, [3, 5, 16, 18, 19, 21] and the references therein. On the other hand, due to the importance of fractional calculus in applications, the study of Lyapunov-type inequalities was extended to fractional boundary value problems by many authors. The first contribution in this direction is due to Ferreira [6], where the fractional boundary value problem

1.5 $$ \begin{aligned} & \bigl(D_{a}^{\alpha}u \bigr) (x)+w(x)u(x)=0,\quad a< x< b, \\ &u(a)=0,\qquad u(b)=0, \end{aligned} $$

with $w\in C([a,b])$, $1<\alpha<2$ and $D_{a}^{\alpha}$ is the Riemann–Liouville fractional derivative of order *α*, was studied. The main result in [6] is the following: If *u* is a nontrivial solution to (1.5), then

1.6 $$ \int_{a}^{b} \bigl\vert w(x) \bigr\vert \,dx> \Gamma(\alpha) \biggl(\frac{4}{b-a} \biggr)^{\alpha-1}. $$

Note that in the limit case $\alpha=2$, (1.5) reduces to (1.2). Moreover, taking $\alpha=2$ in (1.6), we obtain (1.3). For other works related to Lyapunov-type inequalities for fractional boundary value problems, see, for example, [4, 7, 8, 10–12, 20] and the references therein. In particular, in [8], the anti-periodic fractional boundary value problem

1.7 $$ \begin{aligned} & \bigl({}^{C}D_{a}^{\alpha}u \bigr) (x)+w(x)u(x)=0,\quad a< x< b, \\ &u(a)+u(b)=0,\qquad u'(a)+u'(b)=0, \end{aligned} $$

where $w\in C([a,b])$, $1<\alpha<2$ and ${}^{C}D_{a}^{\alpha}$ is the Caputo fractional derivative of order *α*, was studied. Note that (1.7) is a special case of (1.1) with $\psi(x)=x$ and $f(x,z)=w(x)z$.

Motivated by the above cited works, the problem (1.1) is investigated in this paper.

The rest of the paper is organized as follows. In Sect. 2, we recall some basic concepts on fractional calculus and prove some preliminary results. In Sect. 3, a Lyapunov-type inequality is established for problem (1.1). Moreover, some particular cases are discussed. Next, an application to fractional eigenvalue problems is given. In Sect. 4, we end the paper with some open questions.

## Methods and preliminaries

The main idea in this paper consists to reduce (1.1) to a fractional boundary value problem involving Caputo fractional derivative by using an adequate change of variable. Next, using an integral representation of the solution and an estimate of the corresponding Green’s function, a Lyapunov-type inequality is derived for (1.1) under certain assumptions on the functions *f* and *ψ*. Before stating and proving the main results, we need some preliminaries on fractional calculus. The main references used in this part are [2, 13]. For other references related to fractional calculus, see, for example, [1, 14, 15].

First, let us fix $(a,b)\in\mathbb{R}^{2}$ with $a< b$ and $1<\alpha<2$.

Let $\beta>0$. The Riemann–Liouville fractional integral of order *β* of a function $f\in C([a,b])$ is given by (see [13])

$$\bigl(I_{a}^{\beta}f \bigr) (x)=\frac{1}{\Gamma(\beta)} \int_{a}^{x} (x-t)^{\beta-1} f(t)\,dt,\quad a \leq x\leq b, $$

where Γ is the Gamma function.

The Caputo fractional derivative of order *α* of a function $f\in C^{2}([a,b])$ is given by (see [13])

$$\bigl({}^{C}D_{a}^{\alpha}f \bigr) (x)= \bigl(I_{a}^{2-\alpha}f'' \bigr) (x), \quad a< x< b, $$

i.e.,

$$\bigl({}^{C}D_{a}^{\alpha}f \bigr) (x)= \frac{1}{\Gamma(2-\alpha)} \int_{a}^{x} (x-t)^{1-\alpha} f''(t)\,dt,\quad a< x< b. $$

Further, Let $\psi\in C^{2}([a,b])$ be a given function such that

$$\psi'(x)>0,\quad a\leq x\leq b. $$

The fractional integral of order $\beta>0$ of a function $f\in C([a,b])$ with respect to *ψ* is given by (see [13])

$$\bigl(I_{a}^{\beta,\psi}f \bigr) (x)=\frac{1}{\Gamma(\beta)} \int_{a}^{x} \psi '(t) \bigl(\psi(x)- \psi(t) \bigr)^{\beta-1} f(t)\,dt,\quad a\leq x\leq b. $$

The *ψ*-Caputo fractional derivative of order *α* of a function $f\in C^{2}([a,b])$ is given by (see [2])

$$\bigl({}^{C}D_{a}^{\alpha,\psi}f \bigr) (x)= \biggl(I_{a}^{2-\alpha,\psi} \biggl( \frac{1}{\psi'(x)}\frac{d}{dx} \biggr)^{2}f \biggr) (x),\quad a< x< b, $$

i.e.,

$$\bigl({}^{C}D_{a}^{\alpha,\psi}f \bigr) (x)= \frac{1}{\Gamma(2-\alpha)} \int _{a}^{x} \psi'(t) \bigl(\psi(x)- \psi(t) \bigr)^{1-\alpha} \biggl( \frac{1}{\psi'(t)}\frac{d}{dt} \biggr)^{2} f(t)\,dt, \quad a< x< b. $$

The following lemma is crucial for the proof of our main result.

### Lemma 2.1

*Let* $f\in C^{2}([a,b])$. *Then*

$$\bigl({}^{C}D_{a}^{\alpha,\psi}f \bigr) \bigl( \psi^{-1}(y) \bigr)= \bigl({}^{C}D_{\psi(a)}^{\alpha}\bigl(f\circ\psi^{-1} \bigr) \bigr) (y),\quad\psi (a)< y< \psi(b). $$

### Proof

Let $\psi(a)< y< \psi(b)$ be fixed. We have

$$\bigl({}^{C}D_{a}^{\alpha,\psi}f \bigr) \bigl( \psi^{-1}(y) \bigr)= \frac{1}{\Gamma(2-\alpha)} \int_{a}^{\psi^{-1}(y)} \psi'(t) \bigl(y-\psi (t) \bigr)^{1-\alpha} \biggl( \frac{1}{\psi'(t)}\frac{d}{dt} \biggr)^{2} f(t)\,dt. $$

Let us consider the change of variable

$$s=\psi(t),\quad a< t< b. $$

Using the chain rule, we have

$$\frac{d}{ds}=\frac{1}{\psi'(t)}\frac{d}{dt}. $$

Hence, we obtain

$$\bigl({}^{C}D_{a}^{\alpha,\psi}f \bigr) \bigl( \psi^{-1}(y) \bigr)= \frac{1}{\Gamma(2-\alpha)} \int_{\psi(a)}^{y} (y-s)^{1-\alpha} \biggl( \frac {d}{ds} \biggr)^{2} \bigl(f\circ\psi^{-1} \bigr) (s) \,ds, $$

i.e.,

$$\bigl({}^{C}D_{a}^{\alpha,\psi}f \bigr) \bigl( \psi^{-1}(y) \bigr)= \bigl({}^{C}D_{\psi(a)}^{\alpha}\bigl(f\circ\psi^{-1} \bigr) \bigr) (y). $$

 □

We refer the reader to Ferreira [8] for the proofs of the following results.

### Lemma 2.2

*Let* $h\in C([A,B])$, $(A,B)\in\mathbb{R}^{2}$, $A< B$. *Then* $F\in C^{2}([A,B])$ *is a solution to*

$$ \begin{gathered} \bigl({}^{C}D_{A}^{\alpha}F \bigr) (t)+h(t)=0,\quad A< t< B, \\ F(A)+F(B)=0,\,\, F'(A)+F'(B)=0, \end{gathered} $$

*if and only if*

$$F(t)= \int_{A}^{B} (B-s)^{\alpha-2} H(t,s) h(s)\,ds, \quad A\leq t\leq B, $$

*where*

$$\begin{aligned} \Gamma(\alpha)H(t,s)= \textstyle\begin{cases} (\frac{t-A}{2}-\frac{B-A}{4} )(\alpha-1)+\frac{B-s}{2}- \frac{(t-s)^{\alpha-1}}{(B-s)^{\alpha-2}},\quad A\leq s\leq t< B,\\ (\frac{t-A}{2}-\frac{B-A}{4} )(\alpha-1)+\frac{B-s}{2}, \quad A\leq t\leq s\leq B. \end{cases}\displaystyle \end{aligned}$$

### Lemma 2.3

*The function* *H* *defined in Lemma* 2.2 *satisfies*

$$\bigl\vert H(t,s) \bigr\vert \leq\frac{(B-A)(3-\alpha)}{4}, \quad(t,s)\in[A,B] \times[A,B]. $$

## Results and discussion

### A Lyapunov-type inequality for problem (1.1)

In this section, problem (1.1) is investigated under the following assumptions:

(A1): $1<\alpha<2$, $\psi\in C^{2}([a,b])$, $\psi'(x)>0$, $x\in[a,b]$.
(A2): $\psi'(a)=\psi'(b)$.
(A3): The function $f: [a,b]\times\mathbb{R}\to\mathbb{R}$ is continuous and satisfies
  $$\bigl\vert f(x,z) \bigr\vert \leq q(x) \vert z \vert ,\quad(x,z)\in\,]a,b[\, \times\mathbb{R}, $$
  where $q\in C([a,b])$.

Observe that by (A3), we have $f(x,0)=0$, for all $x\in\,]a,b[$. Therefore, 0 is a trivial solution to (1.1).

Our main result is given by the following theorem.

#### Theorem 3.1

*Let* $u\in C^{2}([a,b])$ *be a nontrivial solution to* (1.1). *Then*

3.1 $$ \int_{a}^{b} \bigl(\psi(b)-\psi(x) \bigr)^{\alpha-2} q(x) \psi'(x)\,dx\geq\frac {4}{(\psi(b)-\psi(a))(3-\alpha)}. $$

#### Proof

Let $u\in C^{2}([a,b])$ be a nontrivial solution to (1.1). We introduce the function

$$v: \bigl[\psi(a),\psi(b) \bigr]\to\mathbb{R} $$

given by

3.2 $$ v(y)=u \bigl(\psi^{-1}(y) \bigr),\quad \psi(a)\leq y \leq \psi(b). $$

Using Lemma 2.1, we obtain

$$\bigl({}^{C}D_{\psi(a)}^{\alpha}v \bigr) (y)= \bigl({}^{C}D_{a}^{\alpha,\psi}u \bigr) \bigl( \psi^{-1}(y) \bigr),\quad \psi(a)< y< \psi(b), $$

which implies from (1.1) that

3.3 $$ \bigl({}^{C}D_{\psi(a)}^{\alpha}v \bigr) (y)+f \bigl(\psi^{-1}(y),v(y) \bigr)=0,\quad \psi(a)< y< \psi(b). $$

On the other hand, we have

$$v'(y)=\frac{1}{\psi'(\psi^{-1}(y))} u' \bigl( \psi^{-1}(y) \bigr)),\quad\psi(a)\leq y\leq\psi(b). $$

Therefore,

$$v' \bigl(\psi(a) \bigr)= \frac{1}{\psi'(a)} u'(a) \quad \mbox{and}\quad v' \bigl(\psi(b) \bigr) =\frac{1}{\psi'(b)} u'(b), $$

which implies form (A2) and the boundary conditions in (1.1) that

3.4 $$ v \bigl(\psi(a) \bigr)+v \bigl(\psi(b) \bigr)=0\quad\mbox{and} \quad v' \bigl(\psi(a) \bigr)+v' \bigl(\psi(b) \bigr)=0. $$

Therefore, $v\in C^{2}([A,B])$, $(A,B)=(\psi(a),\psi(b))$, is a nontrivial solution to (3.3)–(3.4). Further, using Lemma 2.2, we obtain

$$v(y)= \int_{A}^{B} (B-s)^{\alpha-2} H(y,s) f \bigl( \psi^{-1}(s),v(s) \bigr)\,ds,\quad A\leq y\leq B. $$

Next, using (A3) and the estimate given by Lemma 2.3, for all $A\leq y\leq B$, we obtain

$$\begin{aligned} \bigl\vert v(y) \bigr\vert \leq& \int_{A}^{B} (B-s)^{\alpha-2} \bigl\vert H(y,s) \bigr\vert \bigl\vert f \bigl(\psi ^{-1}(s),v(s) \bigr) \bigr\vert \,ds \\ \leq& \frac{(B-A)(3-\alpha)}{4} \int_{A}^{B} (B-s)^{\alpha-2} q \bigl(\psi ^{-1}(s) \bigr) \bigl\vert v(s) \bigr\vert \,ds \\ \leq& \biggl(\frac{(B-A)(3-\alpha)}{4} \int_{A}^{B} (B-s)^{\alpha-2} q \bigl(\psi ^{-1}(s) \bigr)\,ds \biggr) \Vert v \Vert _{\infty}, \end{aligned}$$

where

$$\Vert v \Vert _{\infty}=\max \bigl\{ \bigl\vert v(s) \bigr\vert : \, A\leq s\leq B \bigr\} . $$

Since $\|v\|_{\infty}>0$ (because *v* is nontrivial), we obtain

$$\int_{A}^{B} (B-s)^{\alpha-2} q \bigl( \psi^{-1}(s) \bigr)\,ds\geq\frac {4}{(B-A)(3-\alpha)}. $$

Finally, using the change of variable

$$x=\psi^{-1}(s),\quad A\leq s\leq B, $$

inequality (3.1) follows. □

Further, let us discuss some particular cases following from Theorem 3.1.

We consider the case

$$\psi(x)=x,\quad a\leq x\leq b. $$

In this case, problem (1.1) reduces to

3.5 $$ \begin{aligned} & \bigl({}^{C}D_{a}^{\alpha}u \bigr) (x)+f \bigl(x,u(x) \bigr)=0,\quad a< x< b, \\ &u(a)+u(b)=0,\qquad u'(a)+u'(b)=0, \end{aligned} $$

where $1<\alpha<2$. Observe that the function *ψ* satisfies assumptions (A1) and (A2). Therefore, under assumption (A3), from Theorem 3.1, we deduce the following result.

#### Corollary 3.2

*Let* $u\in C^{2}([a,b])$ *be a nontrivial solution to* (3.5). *Then*

$$\int_{a}^{b} (b-x)^{\alpha-2} q(x) \,dx\geq \frac{4}{(b-a)(3-\alpha)}. $$

Next, let us consider the fractional boundary value problem

3.6 $$ \begin{aligned} & \bigl({}^{C}D_{a}^{\alpha}u \bigr) (x)+w(x)u(x)=0,\quad a< x< b, \\ &u(a)+u(b)=0,\qquad u'(a)+u'(b)=0, \end{aligned} $$

where $1<\alpha<2$ and $w\in C([a,b])$. Problem (3.6) is a special case of (3.5) with

$$f(x,z)=w(x) z,\quad (x,z)\in[a,b]\times\mathbb{R}. $$

Observe that the function *f* satisfies assumption (A3) with

$$q(x)= \bigl\vert w(x) \bigr\vert ,\quad a\leq x\leq b. $$

Therefore, by Corollary 3.2, we deduce the following result, which was derived in [8] (with strict inequality).

#### Corollary 3.3

*Let* $u\in C^{2}([a,b])$ *be a nontrivial solution to* (3.6). *Then*

3.7 $$ \int_{a}^{b} (b-x)^{\alpha-2} \bigl\vert w(x) \bigr\vert \,dx\geq\frac{4}{(b-a)(3-\alpha)}. $$

Let us consider the fractional boundary value problem

3.8 $$ \begin{aligned} & \bigl({}^{C}D_{a}^{\alpha}u \bigr) (x)+w(x)\sin \bigl(u(x) \bigr)=0,\quad a< x< b, \\ &u(a)+u(b)=0,\qquad u'(a)+u'(b)=0, \end{aligned} $$

where $1<\alpha<2$ and $w\in C([a,b])$. Problem (3.6) is a special case of (3.5) with

$$f(x,z)=w(x) \sin(z),\quad (x,z)\in[a,b]\times\mathbb{R}. $$

Observe that the function *f* satisfies assumption (A3) with

$$q(x)= \bigl\vert w(x) \bigr\vert ,\quad a\leq x\leq b. $$

Therefore, by Corollary 3.2, we deduce the following result.

#### Corollary 3.4

*Let* $u\in C^{2}([a,b])$ *be a nontrivial solution to* (3.8). *Then* (3.7) *holds*.

Let us consider the fractional boundary value problem

3.9 $$ \begin{aligned} & \bigl({}^{C}D_{a}^{\alpha}u \bigr) (x)+w(x)\arctan \bigl(u(x) \bigr)=0,\quad a< x< b, \\ &u(a)+u(b)=0,\qquad u'(a)+u'(b)=0, \end{aligned} $$

where $1<\alpha<2$ and $w\in C([a,b])$. Problem (3.9) is a special case of (3.5) with

$$f(x,z)=w(x) \arctan(z),\quad (x,z)\in[a,b]\times\mathbb{R}. $$

Note that the function *f* satisfies assumption (A3) with

$$q(x)= \bigl\vert w(x) \bigr\vert ,\quad a\leq x\leq b. $$

Therefore, by Corollary 3.2, we deduce the following result.

#### Corollary 3.5

*Let* $u\in C^{2}([a,b])$ *be a nontrivial solution to* (3.9). *Then* (3.7) *holds*.

Further, we consider the case

3.10 $$ \psi(x)=\frac{x^{2N+1}}{2N+1}+c_{1}x+c_{2}, \quad-1\leq x\leq1, $$

where $N\geq1$ is a natural number, $c_{1}>0$ and $c_{2}\in\mathbb{R}$. Observe that $\psi\in C^{2}([-1,1])$. Moreover, we have

$$\psi'(x)=x^{2N}+c_{1}>0,\quad -1\leq x\leq1. $$

Observe also that

$$\psi'(-1)=\psi'(1)=c_{1}+1. $$

Therefore, the function *ψ* satisfies assumptions (A1) and (A2) with $(a,b)=(-1,1)$. Hence, by Theorem 3.1, we deduce the following result.

#### Corollary 3.6

*Let* $u\in C^{2}([a,b])$ *be a nontrivial solution to* (1.1), *where* $(a,b)=(-1,1)$ *and the function* *ψ* *is given by* (3.10). *Then*

3.11 $$ \int_{-1}^{1} \biggl(\frac{1-x^{2N+1}}{2N+1}+c_{1}(1-x) \biggr)^{\alpha-2} q(x) \bigl(x^{2N}+c_{1} \bigr)\,dx\geq \frac{2}{ (\frac{1}{2N+1}+c_{1} )(3-\alpha)}. $$

Let us consider the case

3.12 $$ \psi(x)=\sinh(x),\quad-1\leq x\leq1. $$

Observe that $\psi\in C^{2}([-1,1])$. Moreover, we have

$$\psi'(x)=\cosh(x)>0,\quad -1\leq x\leq1. $$

Note that due to the parity of the function $\cosh(x)$, we have

$$\psi'(-1)=\cosh(-1)=\cosh(1)=\psi'(1). $$

Therefore, the function *ψ* satisfies assumptions (A1) and (A2) with $(a,b)=(-1,1)$. Hence, by Theorem 3.1, we deduce the following result.

#### Corollary 3.7

*Let* $u\in C^{2}([a,b])$ *be a nontrivial solution to* (1.1), *where* $(a,b)=(-1,1)$ *and the function* *ψ* *is given by* (3.12). *Then*

3.13 $$ \int_{-1}^{1} \bigl(\sinh(1)-\sinh(x) \bigr)^{\alpha-2} q(x) \cosh (x)\,dx\geq\frac{2}{\sinh(1) (3-\alpha)}. $$

Many other results can be deduced from Theorem 3.1 for different choices of functions *f* and *ψ*. We end this section with additional examples of functions *f* and *ψ* satisfying assumptions (A1), (A2) and (A3):

$$\begin{aligned} &\psi(x)=\tan(x), \qquad \vert x \vert \leq \frac{\pi}{4}, \\ &\psi(x)= \arcsin(x),\qquad \vert x \vert \leq \frac{1}{2}, \\ &\psi(x)=\ln \biggl(\frac{1+x}{1-x} \biggr),\qquad \vert x \vert \leq \frac{1}{2}, \\ &\psi(x)= \int_{-1}^{x} e^{s^{2}}\,ds,\qquad \vert x \vert \leq 1, \end{aligned}$$

and

$$\begin{aligned} &f(x,z)=w(x) \cos \biggl(z+\frac{\pi}{2} \biggr), \quad(x,z)\in [a,b] \times\mathbb{R}, \\ &f(x,z)=w(x) ze^{- \vert z \vert }, \quad(x,z)\in[a,b]\times\mathbb{R}, \\ &f(x,z)=\frac{w(x) z}{\cosh(z)}, \quad(x,z)\in[a,b]\times\mathbb {R}, \\ &f(x,z)= w(x) \ln \bigl(1+ \vert z \vert \bigr),\quad(x,z)\in[a,b]\times \mathbb{R}, \end{aligned}$$

where $w\in C([a,b])$.

### An application to eigenvalue problems

Let $\psi\in C^{2}([a,b])$ be a given function satisfying assumptions (A1) and (A2). We say that $\lambda\in\mathbb{R}$ is an eigenvalue of the fractional boundary value problem

3.14 $$ \begin{aligned} & \bigl({}^{C}D_{a}^{\alpha,\psi}u \bigr) (x)+\lambda u(x)=0,\quad a< x< b, \\ &u(a)+u(b)=0,\qquad u'(a)+u'(b)=0, \end{aligned} $$

where $1<\alpha<2$, if and only if (3.14) admits a nontrivial solution $u_{\lambda}\in C^{2}([a,b])$.

The following result provides an upper bound of possible eigenvalues of (3.14).

#### Theorem 3.8

*If* *λ* *is an eigenvalue of* (3.14), *then*

3.15 $$ \vert \lambda \vert \geq\frac{4(\alpha-1)}{(3-\alpha)(\psi(b)-\psi(a))^{\alpha}}. $$

#### Proof

Let $\lambda\in\mathbb{R}$ be an eigenvalue of (3.14). Then (3.14) admits a nontrivial solution $u_{\lambda}\in C^{2}([a,b])$. On the other hand, observe that (3.14) is a special case of (1.1) with

$$f(x,z)=\lambda z,\quad(x,z)\in[a,b]\times\mathbb{R}. $$

Moreover, the function *f* satisfies assumption (A3) with

$$q(x)=\lambda,\quad a\leq x\leq b. $$

Hence, by Theorem 3.1, we obtain

$$\begin{aligned} \vert \lambda \vert \geq& \frac{4}{(\psi(b)-\psi(a))(3-\alpha)} \biggl( \int_{a}^{b} \bigl(\psi(b)-\psi(x) \bigr)^{\alpha-2} \psi'(x)\,dx \biggr)^{-1} \\ =& \frac{4(\alpha-1)}{(3-\alpha)(\psi(b)-\psi(a))^{\alpha}}. \end{aligned}$$

Therefore, we proved (3.15). □

Taking

$$\psi(x)=x,\quad a\leq x\leq b $$

in (3.14), we deduce the following result, which was obtained in [8].

#### Corollary 3.9

*Let* $\lambda\in\mathbb{R}$ *be an eigenvalue of the fractional boundary value problem*

$$\begin{gathered} \bigl({}^{C}D_{a}^{\alpha}u \bigr) (x)+\lambda u(x)=0,\quad a< x< b, \\ u(a)+u(b)=0,\qquad u'(a)+u'(b)=0, \end{gathered} $$

*where* $1<\alpha<2$. *Then*

$$\vert \lambda \vert \geq\frac{4(\alpha-1)}{(3-\alpha)(b-a)^{\alpha}}. $$

## Conclusion

In this paper, a Lyapunov-type inequality is established for the fractional boundary value problem (1.1) under assumptions (A1), (A2) and (A3). Next, the obtained inequality is used to obtain bounds on possible eigenvalues of the corresponding problem. We end the paper with the following open questions. First, it would be interesting to compute the Green’s function for the fractional boundary value problem

$$ \begin{aligned} & \bigl({}^{C}D_{A}^{\alpha}F \bigr) (t)+h(t)=0,\quad A< t< B, \\ &F(A)+F(B)=0,\qquad F'(A)+\mu F'(B)=0, \end{aligned} $$

where $\mu>0$, $h\in C([A,B])$, and to obtain an estimate similar to that given by Lemma 2.3.

Next, the obtained estimate can be used to derive a Lyapunov-type inequality for problem (1.1) by considering a more general class of functions *ψ* without assumption (A2). In fact, from the proof of Theorem 3.1, the function *v* given by (3.2) satisfies (3.3) and the boundary conditions

$$v(A)=v(B)=0\quad\mbox{and}\quad v'(A)+\frac{\psi'(b)}{\psi'(a)} v'(B)=0, $$

where $(A,B)=(\psi(a),\psi(b))$.
